# Segmental Vitiligo: Rapid Spread From a Halo Nevus

**DOI:** 10.7759/cureus.82378

**Published:** 2025-04-16

**Authors:** Monica Z Trevino, Jodie Shao, Elene Valladares, Dale Quest

**Affiliations:** 1 Medicine, Texas Tech University Health Sciences Center El Paso Paul L. Foster School of Medicine, El Paso, USA; 2 Medical Education, Texas Tech University Health Sciences Center El Paso Paul L. Foster School of Medicine, El Paso, USA

**Keywords:** autoimmune and genetic skin diseases, blaschko's lines, dermatology trends, dermatomal pattern, halo nevus, nonsegmental vitiligo, pediatric dermatology, segmental vitiligo, stable vitiligo, vitiligo

## Abstract

Vitiligo, a skin condition characterized by depigmentation, is classified into segmental and non-segmental forms based on onset and distribution. While halo nevi typically accompany non-segmental vitiligo, this report documents a rare case where a halo nevus transitioned into segmental vitiligo in an adolescent male. The depigmentation exhibited a distinct unilateral pattern, halting at the midline, which is characteristic of segmental vitiligo. Unlike non-segmental vitiligo, segmental vitiligo is less commonly associated with autoimmune diseases, and this patient's lack of autoimmune comorbidity aligns with that pattern. The depigmentation followed Blaschko’s lines, which typically do not cross the midline. Based on the consultation at the time, no immediate treatment was recommended. Recent guidelines suggest first-line use of topical calcineurin inhibitors like tacrolimus or JAK/STAT inhibitors like ruxolitinib. Understanding diverse vitiligo presentations is crucial for more precise diagnosis and treatment strategies.

## Introduction

This report presents a case of segmental vitiligo evolving from a halo nevus in an adolescent. A halo nevus is a mole with a surrounding halo of depigmented skin caused by the immune system attacking melanocytes, the cells that give skin its pigment. Melanocyte destruction is correlated with interferon-gamma (IFN-γ) release, therefore activating CXCR3+ CD8+ T cells, triggering melanocyte apoptosis. The nevus will gradually decrease in size and disappear spontaneously, leaving only depigmented skin [1]. This is thought to be a potential signal for the onset of vitiligo. Sometimes, the area will re-pigment outwardly from spared melanocytes in the hair follicle over months or years. Koebner’s phenomenon, observed as a trigger for conditions including vitiligo or psoriasis, manifests in areas where the skin has been injured or irritated [2,3]. As such, sunburns can act as a physical trigger for Koebner's phenomenon, leading to the progression of trauma-induced depigmentation in susceptible individuals [3].

This case was previously presented at the 2nd Annual El Paso Pediatric Colloquium as a clinical conundrum on June 6, 2015.

## Case presentation

**Figure 1:**
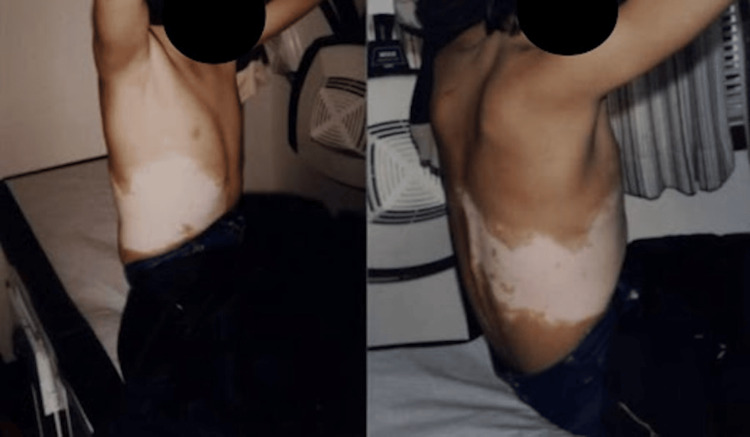
Segmental pattern Spread of segmental depigmentation across the right side of the chest, halting at the midline.

A male adolescent of Athapaskan descent (Dene) initially presented with a single halo nevus at the right fourth intercostal level along the anterior axillary line. The initial and only halo nevus present was a dark mahogany color approximately 2.5 mm in diameter. The depigmented skin around the nevus was around 1.5 cm, displaying a symmetrical, pink-cream hue. After the initial consultation with a senior dermatologist, it was assumed that the depigmented patch would halt and remain quiescent. However, the depigmented patch rapidly evolved over one week across the right chest up to the midline and then remained quiescent, as seen at the time of follow-up from the initial visit (Figure 1). This pattern of spreading is typical for segmental vitiligo but unusual in its association with a halo nevus.

In addition to segmental vitiligo, the patient’s symmetrical pityriasis alba on the cheeks presented as non-defined, hypopigmented patches with slight scaling. The patient's skin condition did not extend beyond the described areas, and there were no other significant dermatological findings. The diagnosis was confirmed visually, as standard diagnostic tools were unavailable in the rural setting of the visit.

The examination revealed no other associated symptoms or signs of systemic illness. The patient was not being treated for any conditions, with no known history or family history of autoimmune diseases, which is a consideration in cases of nonsegmental vitiligo. This case was seen during a locum tenens, and labs were not performed at the time.

## Discussion

**Figure 2:**
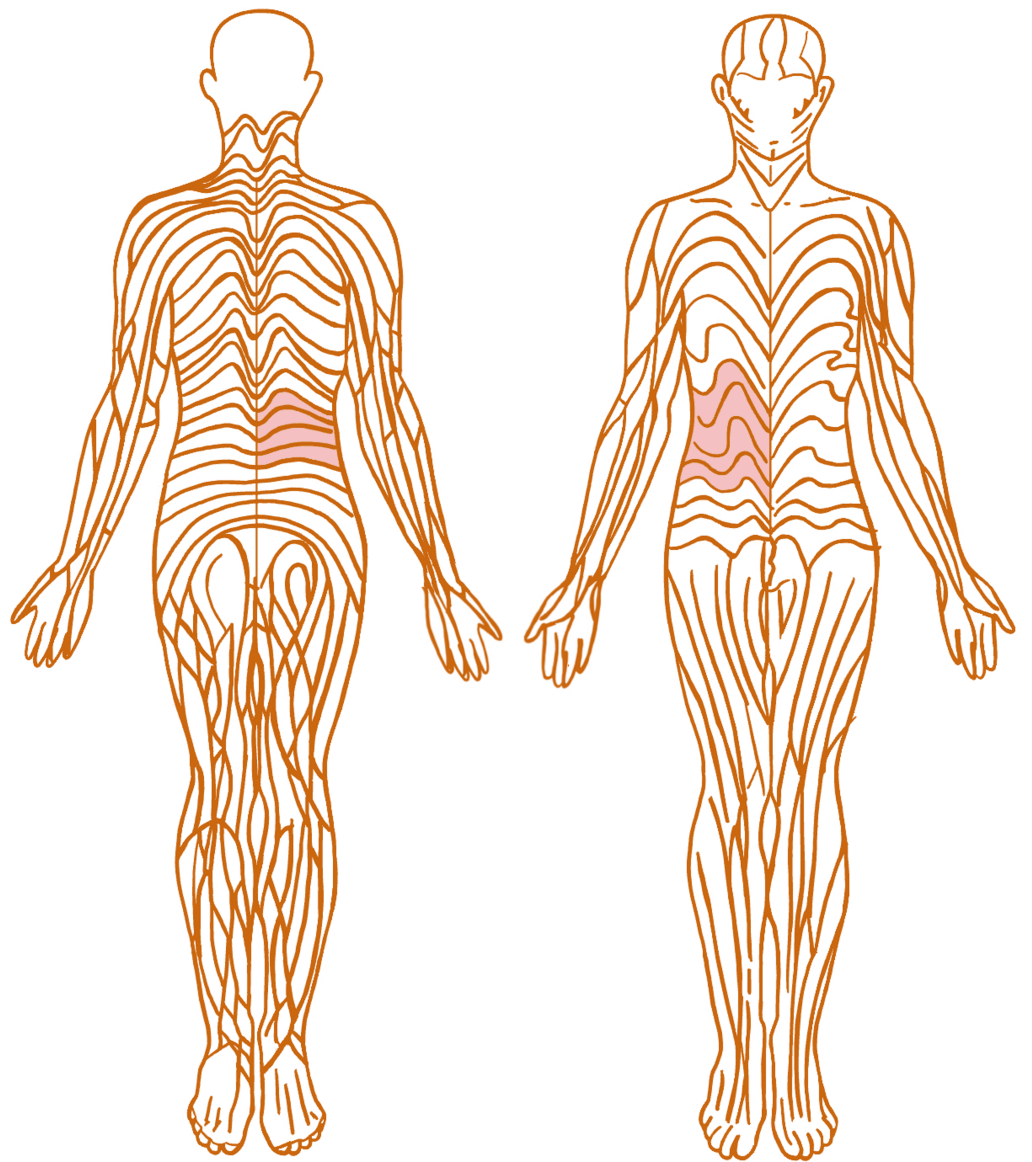
Blashcko's lines The highlighted segments of the figure represent areas of depigmented skin identified on the adolescent male presented in our case. This image is adapted from: Blaschko A. (1901) *Die Nervenverteilung in der Haut in ihrer Beziehung zu den Erkrankungen der Haut*. Retrieved from public domain [11].

Segmental vitiligo spreading from a halo nevus, as seen in our case, is rare and typically follows Blaschko’s lines, not crossing the midline [4-6]. Misattribution is not surprising for this pattern; due to its dermatomal pattern, some suggest it should be termed zosteriform rather than Blaschoid [7]. Blaschko's lines have an arc-like arrangement, as seen in Figure 2, and result from cutaneous mosaicism. Table 1 provides a concise comparison of segmental and non-segmental vitiligo with our case. 

**Table 1:** Comparison of segmental and nonsegmental vitiligo with our case presentation

	Segmental Vitiligo	Nonsegmental Vitiligo	Case Presentation: Segmental Vitiligo From a Halo Nevus
Onset	Childhood or adolescence [7]	Bimodal [5]	Adolescent
Koebner’s phenomenon	Rarely observed [8]	Commonly observed [5]	Not observed
Presence of halo nevi	Uncommon [7]	Common [5]	Observed
Distribution	Unilateral with a sharp demarcation around the midline [6]	Symmetrical well-defined macules crossing midline [5]	Unilateral, halted at the midline
Depigmentation progression	Rapid progression, stabilizes without further spread [6]	Variable, can continue spreading throughout life [5]	Rapid spread in segmental pattern, then quiescent
Autoimmune disease linkages	Less correlation [6,9]	Linked with allergies and other immunological diseases like canities (premature hair graying) or atopy [5]	No known autoimmune diseases
Treatment	Topical therapies, targeted phototherapy, and surgical therapy [10]	Topical therapies, targeted phototherapy, and surgical therapy [10]	No FDA-approved treatment for pediatric vitiligo [10]

Manifestation

**Figure 3:**
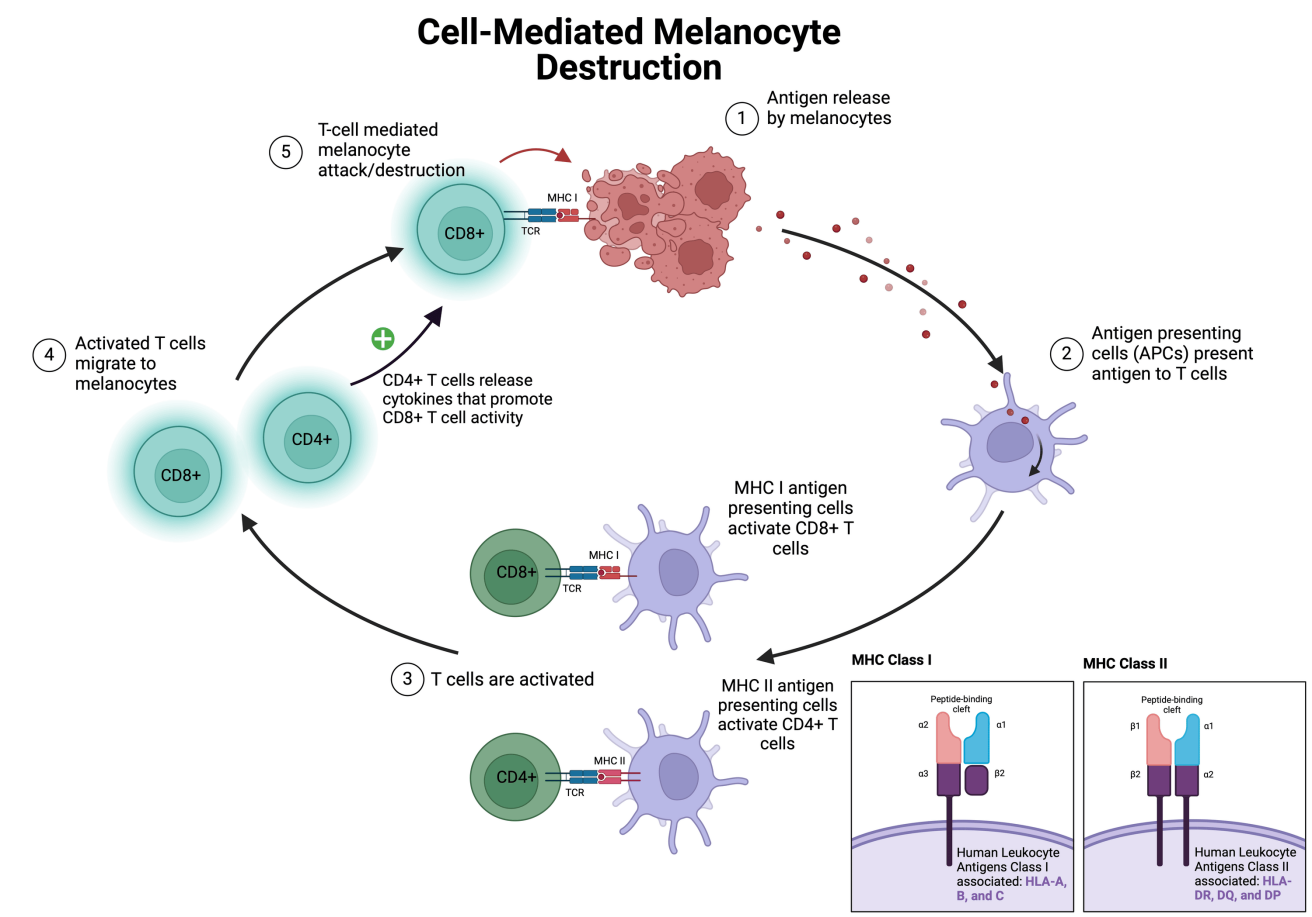
Cell-mediated melanocyte destruction This figure shows the process in which antigen-presenting cells (APCs) identify antigens from melanocytes as foreign material in vitiligo. Diagrams of both major histocompatibility complexes (MHCs) I and II include which human leukocyte antigens (HLA) they are respectively associated with. Adapted from a BioRender template titled "Antigen Presentation in Cancer" [14].

Cutaneous mosaicism refers to the presence of multiple genetic cell populations, some mutated and some not, within the same skin area. Variations in human leukocyte antigen (HLA) genes potentially influence the development of vitiligo by affecting self-antigen presentation to T cells, leading to healthy melanocyte destruction [12]. As illustrated in Figure 3, CD8+ T cells infiltrate the skin and release cytotoxic molecules like granzyme B and perforin, leading to the apoptosis of melanocytes. Interferon-gamma (IFN-γ) is secreted by activated CD8+ T cells, promoting an inflammatory environment through pathways like JAK-STAT and other cytokines, while also inducing the expression of chemokine (C-X-C motif) ligands 9 and 10 to attract more C-X-C chemokine receptors like CXCR3+ T cells to the site of inflammation [10]. CXCR3+ is suggested to have a role in the generation of effector and memory T cells amplifying immune response and trafficking of T cells against melanocytes [10,13].

**Figure 4:**
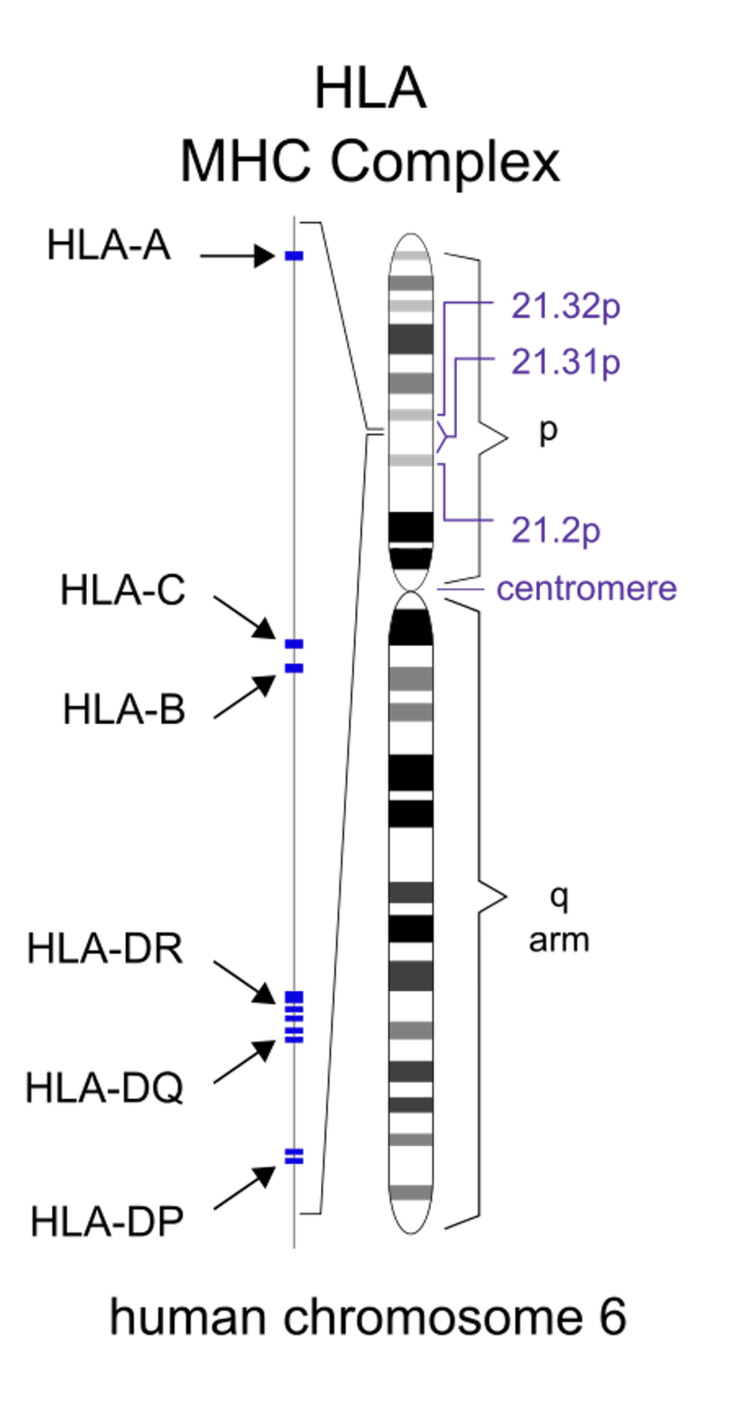
HLA MHC complex on chromosome 6 The human leukocyte antigen (HLA) genes, responsible for encoding major histocompatibility complex (MHC) proteins, are located on chromosome 6. MHC I is associated with the HLA A, B, and C genes. MHC II is associated with the HLA DR, DQ, and DP genes. Sourced from Wikimedia Commons [15].

HLA genes, part of major histocompatibility complexes (MHCs), are crucial in distinguishing self from foreign material in the immune system, as seen in Figure 4.

Follow-up with audiologists and ophthalmologists is recommended due to potential melanocyte destruction in the inner ear and iris, causing sensorineural hearing loss or uveitis [16]. Specific HLA genetic expressions, such as HLA-DR4, correlate with different vitiligo manifestations [17]. Patients with segmental vitiligo linked to halo nevi likely possess HLA genes, reducing their risk of developing other autoimmune diseases, explaining the lower incidence of autoimmune diseases in segmental vitiligo compared to non-segmental forms [12].

Treatment

At the time of the visit, there were no immediate treatment recommendations. The spread of the depigmented patch after the first visit was unpredictable. According to the guidelines, the first-line use of topical calcineurin inhibitors in pediatric vitiligo, like tacrolimus, has shown effectiveness for depigmentation on the head and neck with intermediate response on the trunk and extremities [10]. JAK/STAT inhibitors like ruxolitinib in patients 12 years and older have been FDA-approved since 2022 for vitiligo affecting less than 10% of the body surface area [10]. Ruxolitinib is a JAK/STAT inhibitor and therefore prevents CD8+ T cell recruitment for melanocyte destruction. Narrow-band ultraviolet B (NB-UVB) phototherapy is used for larger affected areas or can be used in combination with topical calcineurin inhibitors [18].

## Conclusions

This case of an adolescent developing segmental vitiligo from an initial halo nevus is noteworthy. Typically, segmental vitiligo is not linked to the presence of a halo nevus as much as it is associated with nonsegmental vitiligo. Recognizing diverse manifestations of vitiligo may promote earlier treatment interventions. Increasing awareness of atypical presentations might lead to improved management strategies and outcomes for patients with vitiligo.

## References

[1] Bergqvist C, Ezzedine K (2020). Vitiligo: a review. Dermatology.

[2] Delbaere L, van Causenbroeck J, Duponselle J, Van Goethem C, Speeckaert R, van Geel N (2024). Hot spots for clinical signs of disease activity in vitiligo. Exp Dermatol.

[3] Jin Y, Santorico SA, Spritz RA (2020). Pediatric to adult shift in vitiligo onset suggests altered environmental triggering. J Invest Dermatol.

[4] Cohen BE, Mu EW, Orlow SJ (2016). Comparison of childhood vitiligo presenting with or without associated halo nevi. Pediatr Dermatol.

[5] Taïeb A, Picardo M (2009). Clinical practice. Vitiligo. N Engl J Med.

[6] Speeckaert R, Lambert J, Bulat V, Belpaire A, Speeckaert M, van Geel N (2020). Autoimmunity in segmental vitiligo. Front Immunol.

[7] van Geel N, De Lille S, Vandenhaute S, Gauthier Y, Mollet I, Brochez L, Lambert J (2011). Different phenotypes of segmental vitiligo based on a clinical observational study. J Eur Acad Dermatol Venereol.

[8] Martins CP, Hertz A, Luzio P, Paludo P, Azulay-Abulafia L (2020). Clinical and epidemiological characteristics of childhood vitiligo: a study of 701 patients from Brazil. Int J Dermatol.

[9] Majid I, Imran S (2020). Excimer light therapy in childhood segmental vitiligo: early treatment gives better results. Dermatol Ther.

[10] Groom JR, Luster AD (2011). CXCR3 in T cell function. Exp Cell Res.

[11] Blaschko A (1901). Tafel XVI. Die Nervenverteilung in der Haut in ihrer Beziehung zu den Erkrankungen der Haut [In German].

[12] de Vijlder HC, Westerhof W, Schreuder GM, de Lange P, Claas FH (2004). Difference in pathogenesis between vitiligo vulgaris and halo nevi associated with vitiligo is supported by an HLA association study. Pigment Cell Res.

[13] Renert-Yuval Y, Ezzedine K, Grimes P (2024). Expert recommendations on use of topical therapeutics for vitiligo in pediatric, adolescent, and young adult patients. JAMA Dermatol.

[14] Trevino M: Cell Mediated Melanocyte Destruction (2024). Cell mediated melanocyte destruction adapted from “Antigen presentation in cancer”. Biorender. BioRender.com.

[15] contributors WC: File:HLA.svg (2024). File:HLA.svg. Wikimedia Commons.

[16] Ma SH, Ang MD, Chang YT, Dai YX (2021). Association between vitiligo and hearing loss. J Am Acad Dermatol.

[17] Oliveira-Caramez ML, Veiga-Castelli L, Souza AS (2023). Evidence for epistatic interaction between HLA-G and LILRB1 in the pathogenesis of nonsegmental vitiligo. Cells.

[18] Tavoletti G, Avallone G, Conforti C (2023). Topical ruxolitinib: a new treatment for vitiligo. J Eur Acad Dermatol Venereol.

